# Lipid metabolism gene-wide profile and survival signature of lung adenocarcinoma

**DOI:** 10.1186/s12944-020-01390-9

**Published:** 2020-10-13

**Authors:** Jinyou Li, Qiang Li, Zhenyu Su, Qi Sun, Yong Zhao, Tienan Feng, Jiayuan Jiang, Feng Zhang, Haitao Ma

**Affiliations:** 1 Department of Thoracic Surgery, Affiliated Hospital of Jiangnan University, Wuxi, 214000 China; 2 Department of Thoracic Surgery, First Affiliated Hospital of Soochow University, Suzhou, 215006 China; 3 Public Health School, Shanghai Jiao Tong University School of Medicine, Shanghai, 200025 China; 4 Clinical Research Institute, Shanghai Jiao Tong University School of Medicine, Shanghai, 200025 China

**Keywords:** Diagnosis, Hub genes, Lipid metabolism, Lung adenocarcinoma, Nomogram, Prognosis, Signature

## Abstract

**Background:**

Lung cancer has high morbidity and mortality across the globe, and lung adenocarcinoma (LUAD) is the most common histologic subtype. Disordered lipid metabolism is related to the development of cancer. Analysis of lipid-related transcriptome helps shed light on the diagnosis and prognostic biomarkers of LUAD.

**Methods:**

In this study, expression analysis of 1045 lipid metabolism-related genes was performed between LUAD tumors and normal tissues derived from the Cancer Genome Atlas Lung Adenocarcinoma (TCGA-LUAD) cohort. The interaction network of differentially expressed genes (DEGs) was constructed to identify the hub genes. The association between hub genes and overall survival (OS) was evaluated and formed a model to predict the prognosis of LUAD using a nomogram. The model was validated by another cohort, GSE13213.

**Results:**

A total of 217 lipid metabolism-related DEGs were detected in LUAD. Genes were significantly enriched in glycerophospholipid metabolism, fatty acid metabolic process, and eicosanoid signaling. Through network analysis and cytoHubba, 6 hub genes were identified, including *INS*, *LPL*, *HPGDS*, *DGAT1*, *UGT1A6*, and *CYP2C9*. High expression of *CYP2C9*, *UGT1A6*, and *INS*, and low expressions of *DGAT1*, *HPGDS*, and *LPL*, were associated with worse overall survival for 1925 LUAD patients. The model showed that the high-risk score group had a worse OS, and the validated cohort showed the same result.

**Conclusions:**

In this study, a signature of 6 lipid metabolism genes was constructed, which was significantly associated with the diagnosis and prognosis of LUAD patients. Thus, the gene signature can be used as a biomarker for LUAD.

## Background

Lung cancer is the most commonly diagnosed cancer (11.6% of the total cases) and the leading cause of cancer-related death (18.4% of the total cancer-related deaths) in the world [1]. Among lung cancer subtypes, adenocarcinoma is the most common histologic subtype of lung cancer in both men and women [2]. In a study published in 2020 in China, it was reported that in the recent 10 years, the percentage of lung adenocarcinoma (LUAD) increased significantly with a decrease in squamous carcinoma [3]. The increasing incidence of lung adenocarcinoma (LUAD) has also been reported to be associated with air pollution-related factors [4–6]. In previous studies, it was reported that PM_2.5_ increased the pro-inflammatory lipid metabolism in the lung and was associated with lipid alterations [7, 8]. The importance of alterations related to lipid metabolism is starting to be recognized, and the increase in de novo lipogenesis has considered a new hallmark in many aggressive cancers [9]. Lipid metabolism has been reported to be associated with many types of cancers, including pancreatic, hepatic, and colorectal cancer [10–12]. Cancer cells exhibit strong lipid and cholesterol uptake. Excess lipids and cholesterol in cancer cells are stored in lipid droplets (LDs) [13, 14]. LDs have been found in lung cancer cells [15]. Moreover, in a previous study, it was reported that some lipid-related phenotypic indices were associated with non-small cell lung cancer (NSCLC). Lipid profiles of blood plasma exosomes were used for the early detection of the prevalent NSCLC [16]. Epidemiological data indicated that a certain number of lung cancer patients with high high-density lipoprotein cholesterol (HDL-C) and low-density lipoprotein (LDL) and low-density lipoprotein receptor (LDLR) levels had better survival rates in patients [17, 18]. Compared with healthy subjects, NSCLC patients showed significant increases in levels of phosphatidylcholine (PC) and phosphatidylethanolamine (PE) [19]. Other lipid metabolism indicators associated with LUAD include sphingomyelins, phosphatidylinositols, phosphatidylserines, phosphatidylethanolamine, phospholipids, and phosphatidylcholine [20]. The requirement of cancer cells for metabolic intermediates for macromolecule production is overwhelming. Fatty acid oxidation (FAO) can help generate ATP to support the membranes formation, energy storage, production of signaling molecules by coordinating the activation of lipid anabolic metabolism [21]. Regulation of the lipid metabolism to LUAD is still being explored, and identifying the underlying lipid-related mechanism of the LUAD phenotype will help increase clinical interventions.

To explore the lipid metabolism related to regulatory networks and pathways, an integrated bioinformatic method was used to construct a transcript-wide profile, and a signature of lipid-related genes was analyzed to explore the potential biomarkers for diagnosis and prognostic value of LUAD in terms of lipid metabolism disorder.

## Materials and methods

### Patients and datasets

From 519 LUAD tissues and 58 normal tissues, mRNA expression data and clinical information were downloaded from The Cancer Genome Atlas (TCGA, https://cancergenome.nih.gov/) database using the R package TCGAbiolinks [22]. The ensemble ID of TCGA samples was annotated with human genes GRCh38/hg38. To validate the availability of the final prediction model, mRNA expression data and clinical information from 117 LUAD patients were downloaded from the Gene Expression Omnibus (GEO) database (http://www.ncbi.nlm.nih.gov/geo) (GSE13213) using the R package GEOquery [23].

### Identification of lipid metabolism-related differentially-expressed genes

After using lipid-specific keywords (fatty acyls, glycerolipids, glycerophospholipids, sphingolipids, sterol lipids, prenol lipids, saccharolipids, and polyketides), 21 lipid metabolism-related pathways and five lipid metabolism-related gene sets were collected from the Kyoto Encyclopedia of Genes and Genomes (KEGG) website (http://www.kegg.jp/blastkoala/) [24] and the Molecular Signatures Database (MisDB) website (https://www.gsea-msigdb.org/gsea/msigdb/index.jsp) [25], respectively (Additional file 1). After removing overlapping genes, a total of 1045 lipid metabolism-related genes were obtained. Lipid metabolism-related differentially expressed genes (DEGs) between LUAD tissues and normal tissues were screened through R package edgeR [26]. The parameters set for differential expression analysis were false discovery rate (FDR) < 0.05 and |log2 fold change| (logFC) > 1.

### Bioinformatic analysis

The R package clusterProfiler was used to further explore the biological significance of lipid metabolism-related DEGs [27]. In GO and KEGG analysis, FDR < 0.05 was considered a significant enrichment. Next, DEGs containing gene identifiers and corresponding FDR values and logFC values were uploaded into the IPA software (Qiagen, Redwood City, CA, USA). The “core analysis” function included in the software was used to interpret the DEGs.

### Interaction network generation and hub genes analysis

An interaction network of differentially-expressed lipid metabolism-related genes was built using the Search Tool for the Retrieval of Interacting Genes (STRING, http://string-db.org/) database [28]. The combined score of ≥0.4 was the cut-off value. Cytoscape software (version 3.6.0) was used to visualize networks [29]. According to 12 ranking methods in cytoHubba [30], an APP in Cytoscape, the top ten genes of each method were selected for analysis of overlapping genes, and genes with the highest number of overlaps were used as hub genes and potential biomarkers.

### The expression level analysis of hub genes

The differences in mRNA expression of hub genes between LUAD tissues and normal tissues were verified using the Gene Expression Profiling Interactive Analysis (GEPIA) (http://gepia.cancer-pku.cn/index.html) [31] and ONCOMINE (http://www.oncomine.org) websites. Gene correlation analysis for hub genes was performed using GEPIA.

### Survival analysis

OS analysis of hub genes was employed by Kaplan-Meier Plotter (http://kmplot.com/analysis/), and included clinical data and gene expression information from 719 LUAD patients from GEO and TCGA database [32]. Subsequently, information on the number of cases along with median values of mRNA expression levels, the hazard ratio (HR) with a 95% confidence intervals (CI), and log-rank *P*-values were extracted from the Kaplan-Meier Plotter webpage. Log-rank *P*-values < 0.05 were considered statistically significant.

### Prediction model

Based on the selected hub genes, the nomogram package of R (“rms”) [33] was used to develop a model to evaluate the prognosis of TCGA-LUAD patients. Using the formula of the nomogram, the prognosis score was calculated for each patient. Based on this score, and using the median classification method, patients were divided into a low-risk score group and a high-risk score group. The prognosis score was validated by the patients’ actual prognosis outcome. To investigate whether the expressions of six genes and prognosis score could be independent factors for OS, multivariate Cox regression analysis was performed, including gender, tumor stage, age, and smoking status. Next, expression data of hub genes and clinical information of 117 LUAD patients were downloaded from a different data set (GSE13213), and calculated the prognosis score of each patient by using the formula of the nomogram. Next, patients were divided into two groups, and survival analysis was performed to validate the availability of this model.

## Results

### Identification and functional analysis of lipid metabolism-related DEGs

**Fig. 1:**
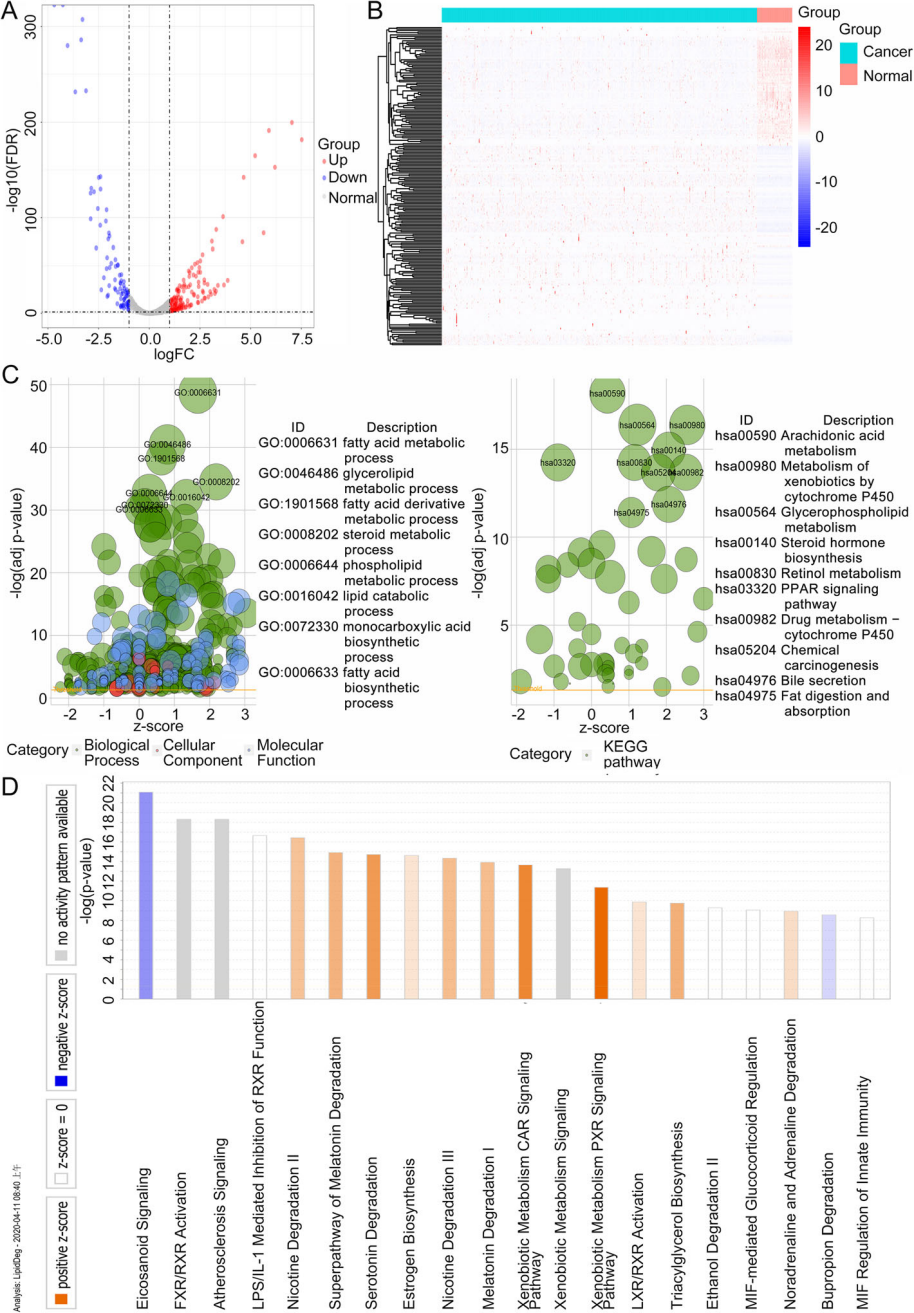
Identification and functional analysis of lipid metabolism-related DEGs. **a** Volcano plot of lipid metabolism-related genes, **b** Heatmap analysis of lipid metabolism-related DEGs, **c** GO and KEGG pathway enrichment analysis by clusterProfiler, **d** functional and signaling pathway enrichment by IPA. In **a** and **b**, red, white, and blue represent higher expression levels, no expression differences, and lower expression levels, respectively

A total of 217 lipid metabolism-related DEGs were identified from the TCGA-LUAD cohort. A volcano plot was constructed to reveal significant DEGs (Fig. 1a), and a heatmap was created to show the hierarchical clustering analysis of the DEGs (Fig. 1b). For the overall understanding of 217 lipid metabolism-related DEGs, GO terms and KEGG pathway enrichment analysis were conducted using the clusterProfiler package, while canonical pathways analysis was performed by IPA. The results of KEGG pathway enrichment showed that DEGs were significantly enriched in arachidonic acid metabolism, metabolism of xenobiotics by cytochrome P450, glycerophospholipid metabolism, and steroid hormone biosynthesis. In contrast, GO terms analysis showed that genes were significantly enriched in the fatty acid metabolic process, glycerolipid metabolic process, fatty acid derivative metabolic process, and steroid metabolic process (Fig. 1c). The genes in each KEGG pathway and GO term are presented in Additional file 2. IPA identified significant canonical networks associated with the DEGs. IPA showed that the top canonical pathways associated with common DEGs including eicosanoid signaling, FXR/RXR activation, and atherosclerosis signaling (Fig. 1d). Combining the results of the three functional analyses showed that DEGs mainly overlapped in glycerophospholipid and steroid metabolism. Furthermore, non-overlapping pathways provided additional information indicating further exploration of the role of lipid metabolism in LUAD.

### Interaction network construction and cytoHubba analysis

**Fig. 2:**
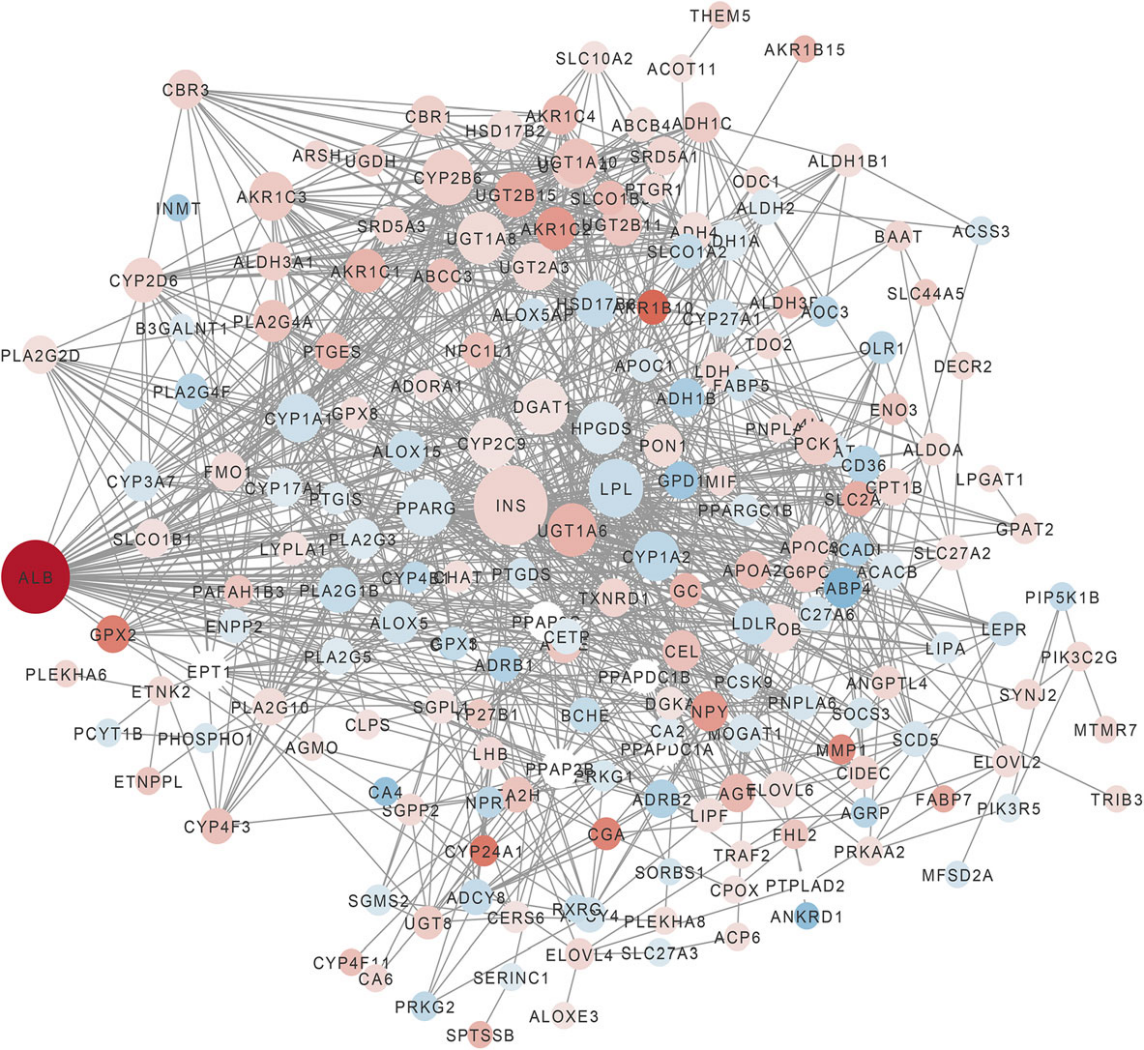
Genes interaction network of lipid metabolism-related DEGs. Red, white, and blue nodes represent upregulated genes, no expression differences genes, and downregulated genes, respectively. The magnitude of the degree is positively correlated with the size of a node

Lipid metabolism-related DEGs were analyzed by the STRING tool. Ultimately, an interaction network with 216 nodes and 1140 edges was established and visualized in Cytoscape (Fig. 2). According to 12 ranked methods in cytoHubba, 6 hub genes were identified by the overlap of the top 10 genes (Additional file 3). Moreover, these genes were related to Insulin (*INS*), Lipoprotein Lipase (*LPL*), Hematopoietic Prostaglandin D Synthase (*HPGDS*), Diacylglycerol O-Acyltransferase 1 (*DGAT1*), UDP Glucuronosyltransferase Family 1 Member A6 (*UGT1A6*), and Cytochrome P450 Family 2 Subfamily C Member 9 (*CYP2C9*).

### The expression level analysis of hub genes

DEG results of hub genes are presented in Table 1. The data showed that *CYP2C9*, *UGT1A6*, *INS*, and *DGAT1* were upregulated, while *HPGDS* and *LPL* were downregulated in TCGA-LUAD tissues compared to normal tissues. To verify the expression results of hub genes, GEPIA and ONCOMINE databases were used. In GEPIA databases, *HPGDS* and *LPL* were significantly downregulated in LUAD samples (Fig. S1). In addition, correlation analysis showed that *LPL* and *DGAT1* (*r* = 0.15; *P* < 0.01), *UGT1A6* and *HPGDS* (*r* = − 0.11; *P* = 0.02), and *HPGDS* and *DGAT1* (*r* = − 0.09; *P* < 0.05) were significantly correlated (Additional file 4). Meta-analysis of 6 hub genes of lung cancer was performed by ONCOMINE databases, and showed that *UGT1A6* and *DGAT1* were upregulated, while *HPGDS* and *LPL* were downregulated (Fig. S2).

**Table 1:** DEG results of hub genes

	logFC	*P*	FDR
*CYP2C9*	1.027946	0.001961	0.004128
*UGT1A6*	3.382161	4.80E-31	6.74E-30
*INS*	1.773607	2.42E-07	8.55E-07
*DGAT1*	1.041643	4.93E-14	3.24E-13
*HPGDS*	−1.19395	6.18E-18	5.18E-17
*LPL*	−1.96376	5.62E-83	2.01E-81

### Survival analysis of hub genes

**Fig. 3:**
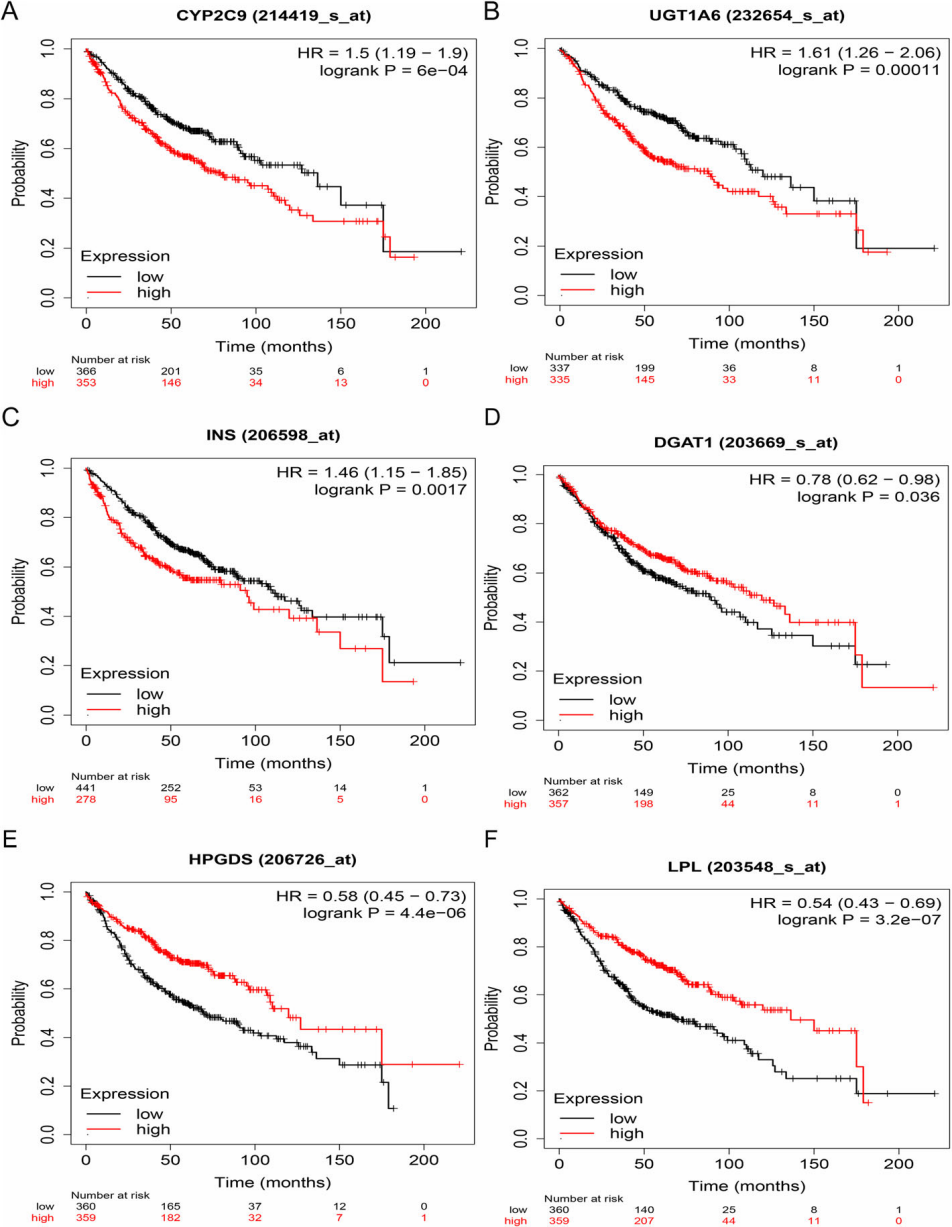
Survival analysis of hub genes. LUAD patients were subdivided into high/low gene expression groups based on the median expression level of each gene in LUAD tissues. **a** OS analysis of *CYP2C9*, **b** OS analysis of *UGT1A6*, **c** OS analysis of *INS*, **d** OS analysis of *DGAT1*, **e** OS analysis of *HPGDS*, and **f** OS analysis of *LPL*

In this study, the relationship between mRNA expression of hub genes and clinical outcome was examined using the Kaplan-Meier plotter. The results showed that high expression of *CYP2C9* [HR = 1.50 (1.19–1.90), *P* < 0.01], *UGT1A6* [HR = 1.61 (1.26–2.06), *P* < 0.01], and *INS* [HR = 1.46 (1.15–1.85), *P* < 0.01], and low expression of *DGAT1* [HR = 0.78 (0.62–0.98), *P* = 0.04], *HPGDS* [HR = 0.58 (0.45–0.73), *P* < 0.01], and *LPL* [HR = 0.54 (0.43–0.69), *P* < 0.01], were associated with a worse OS for 719 LUAD patients (Fig. 3).

### Prediction model based on survival-related hub genes and validation

**Fig. 4:**
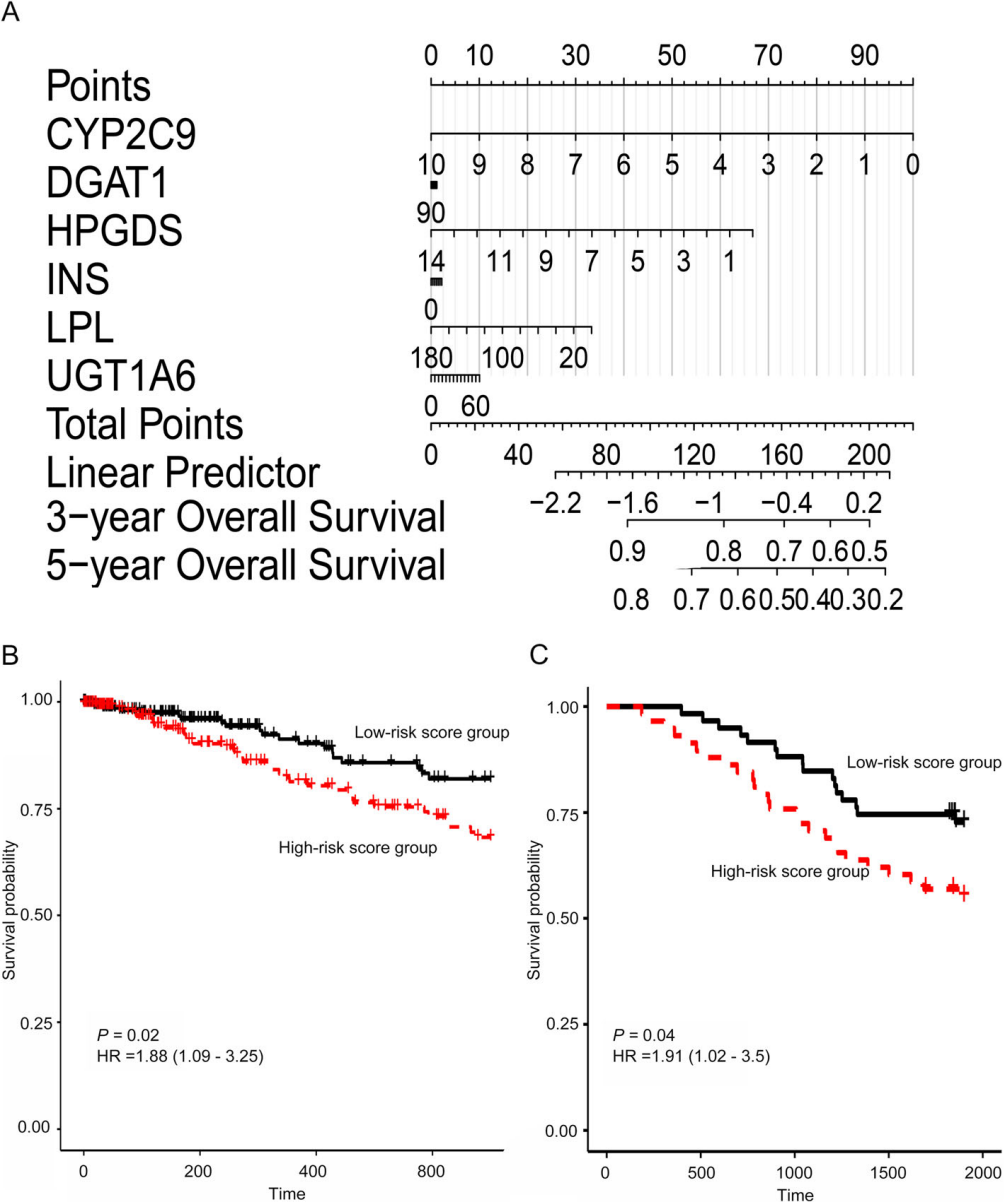
Prediction model based on survival-related hub genes and validation. **a** Nomogram of 6 survival-related genes, **b** survival analysis between the high-risk score group and low-risk score group, and **c** validation of the model

Based on the Cox regression model, a nomogram was built to predict the prognosis of TCGA-LUAD patients, using mRNA expression of the six survival-related hub genes (*CYP2C9*, *DGAT1*, *UGT1A6*, *INS*, *HPGDS*, and *LPL*) (Fig. 4a). The concordance index of the nomogram was 0.61. Subsequently, the prognosis score of each patient was calculated, which showed that patients in the high-risk score group had a worse OS of 3 years [HR = 1.88 (1.09–3.25), *P =* 0.02] (Fig. 4b). A total of 486 patients with complete information, including gender, tumor stage, age, and smoking status were included for the multivariate Cox regression analysis. Except for *HPGDS and LPL*, the HR of *CYP2C9, DGAT1, UGT1A6,* and *INS* was not significant*.* In addition, the risk score calculated from the six-gene signature was an independent prognostic factor (Fig. S3). The model was validated and demonstrated that patients in the high-risk score group had a worse OS [HR = 1.91 (1.02–3.50), *P* = 0.04] (Fig. 4c).

## Discussion

Metabolic changes have been widely observed in a variety of cancer cells [34]. Among the metabolisms involved, the lipid metabolism widely participated in the regulation of many cellular processes, including cell growth, proliferation, differentiation, survival, apoptosis, inflammation, motility, membrane homeostasis, chemotherapy response, and drug resistance [35]. In several recent studies, some components of PM2.5 have been reported as risk factors of lung cancer [36–38], because the PM2.5 components promoted pulmonary injury by modifying lipid metabolism [7] and might be involved in the development of lung cancer. However, studies on the association between lipid metabolism and lung cancer regarding transcriptome-wide analysis are limited. In this study, a LUAD cohort was used to generate a transcriptome-wide profile that included 217 lipid-related genes. The enrichment biological pathway found in LUAD patients included fatty acids, glycerolipids, and glycerophospholipids and were the primary driving enrichment biological function reported [39]. Furthermore, arachidonic acid metabolism, PPAR signaling pathway, insulin resistance, eicosanoids signaling, and other pathways have also been reported in cancer [40–44].

The results indicated that LUAD-related lipid metabolism was associated with nicotine, estrogen biosynthesis, melatonin, and atherosclerosis. Similar to PM2.5, nicotine may promote LUAD development by regulating disordered lipid metabolism. The interaction between estrogen biosynthesis and lipid metabolism may be one of the high-risk factors for LUAD, and is consistent with the observation that LUAD incidence is rising in women, and that the incidence rate among women was higher than that among men [2]. Lipid and cancer-related genes have been shown to be enriched in atherosclerosis and cancer [45]. For the long-term survival of LUAD patients, their health management should be managed by oncologists and cardiologists.

The network of genes was constructed and identified six hub genes related to lipid metabolism and LUAD. *CYP2C9*, a drug target in lung cancer, can inhibit the occurrence of lung cancer by acting on cytochrome P450, thereby regulating tumorigenesis [46, 47]. LUAD patients with a lower expression of *CYP2C9* have a better prognosis than those with a higher expression of *CYP2C9*. *UGT1A* variants may play a minor role in the risk of other types of lung cancer [48]. LUAD patients with a lower expression of *UGT1A6* have a better prognosis than one those with a higher expression of *UGT1A6*. *DGAT1* catalyzes the final step in triglyceride synthesis [49], and *LPL* is a key lipolytic enzyme that plays a crucial role in the catabolism of triglycerides in triglyceride-rich particles [50]. Both are involved in triglyceride synthesis, and triglycerides combined with *HPGDS* have been reported to have therapeutic potential in allergic inflammation [51]. Serum triglyceride concentrations were reported to be involved in the pathogenesis of lung cancer [52]. *INS* encodes insulin and plays a vital role in the regulation of carbohydrate and lipid metabolism. LUAD patients with a lower expression of *INS* have a better prognosis. The regulation of triglyceride synthesis, insulin, and inflammatory control may be an effective intervention of LUAD patients. Based on those six genes, *CYP2C9*, *DGAT1*, *UGT1A6*, *INS*, *HPGDS*, and *LPL*, a risk model was constructed, including that LUAD patients from two cohorts with a lower risk score had a better prognosis.

### Study strengths and limitations

The main strength of the study is the establishment of a lipid metabolic transcriptome-wide profile of LUAD and a gene signature that is significantly associated with the diagnosis and prognosis of LUAD patients. Limitations of this study include the following: 1) The data field information of these two cohorts is limited. Therefore, covariables related to LUAD might be missed and caused bias; 2) The internal mechanism of the six lipid-related genes is not illuminated clearly. In the future, a well-designed study based on the results is warranted.

## Conclusions

In summary, a lipid metabolic transcriptome-wide profile of LUAD patients was generated and showed that lipid metabolic pathways were correlated with LUAD. A signature of six lipid metabolic genes was significantly associated with the diagnosis and prognosis of LUAD patients. Taken together, this gene signature can be used as a biomarker for LUAD to guide the prevention of the occurrence of LUAD and improve the prognosis of LUAD patients.

## Supplementary information

**Additional file 1:.**

**Additional file 2:.**

**Additional file 3: Table S1**. Hub genes for lipid metabolism-related DEGs ranked in cytoHubba.

**Additional file 4: Table S2**. Correlation analysis of expression levels of hub genes in LUAD by GEPIA.

**Additional file 5: Figure S1.** Expression level analysis of 6 hub genes in GEPIA databases. Red and gray represent cancer and normal, respectively. (A) CYP2C9, (B) UGT1A6, (C) INS, (D) DGAT1, (E) HPGDS, and (F) LPL. * *P* < 0.05.

**Additional file 6: Figure S2**. Meta-analysis of 6 hub genes of lung cancer in ONCOMINE databases.

**Additional file 7: Figure S3.** Forrest plot of the multivariate Cox regression analyzis in TCGA-LUAD. (A) Risk score, (B) CYP2C9, (C) UGT1A6, (D) INS, (E) DGAT1, (F) HPGDS, and (G) LPL.

## Data Availability

The datasets generated and/or analyzed during the current study are available in the TCGA & GEO databases, [https://cancergenome.nih.gov/] & [https://www.ncbi.nlm.nih.gov/geo/].

## Abbreviations

LUAD: Lung adenocarcinoma
TCGA-LUAD: The Cancer Genome Atlas Lung Adenocarcinoma
HDL-C: High-density lipoprotein cholesterol
LDL: Low-density lipoprotein
LDLR: Low-density lipoprotein receptor
PC: Phosphatidylcholine
PE: Phosphatidylethanolamine
FAO: Fatty acid oxidation
NSCLC: Non-small-cell lung carcinoma
TCGA: The Cancer Genome Atlas
KEGG: Kyoto Encyclopedia of Genes and Genomes
MisDB: Molecular Signatures Database
DEGs: Differentially expressed genes
FDR: False discovery rate
logFC: Log2 fold change
STRING: Search Tool for the Retrieval of Interacting Genes
OS: Overall survival
HR: Hazard ratio
CI: Confidence interval
*INS*: Insulin
*LPL*: Lipoprotein Lipase
*HPGDS*: Hematopoietic Prostaglandin D Synthase
*DGAT1*: Diacylglycerol O-Acyltransferase 1
*UGT1A6*: UDP Glucuronosyltransferase Family 1 Member A6
*CYP2C9*: Cytochrome P450 Family 2 Subfamily C Member 9
RXRα: Retinoid X receptor alpha
FXRE: FXR response element
