# Supplementary material for: Lipid metabolism gene-wide profile and survival signature of lung adenocarcinoma
Source: Lipids Health Dis. 2020 Oct 13;19:222. doi: 10.1186/s12944-020-01390-9 (PMC7557101; doi:10.1186/s12944-020-01390-9)
Supplement: Supplementary file 3 — Additional file 3: Table S1. Hub genes for lipid metabolism-related DEGs ranked in cytoHubba. [file 12944_2020_1390_MOESM3_ESM.docx]

Table S1. Hub genes for lipid metabolism-related DEGs ranked in cytoHubba

| Catelogy | Rank methods in cytoHubba | | | | | | | | | | | |
| --- | --- | --- | --- | --- | --- | --- | --- | --- | --- | --- | --- | --- |
|  | MCC | DMNC | MNC | Degree | EPC | BottleNeck | EcCentricity | Closeness | Radiality | Betweenness | Stress | ClusteringCoefficient |
| Gene symbol top 10 | UGT1A8 | APOC1 | INS | INS | ALB | INS | INS | INS | INS | INS | INS | ODC1 |
|  | UGT1A6 | SRD5A3 | ALB | ALB | INS | ALB | ALB | ALB | ALB | ALB | ALB | SGMS2 |
|  | AKR1C3 | SRD5A1 | LPL | LPL | CYP2B6 | CEL | CEL | LPL | PPARG | DGAT1 | DGAT1 | CPOX |
|  | AKR1C2 | CETP | CYP2B6 | PPARG | UGT1A8 | LPL | LPL | PPARG | LPL | PPARG | CYP2B6 | DECR2 |
|  | HSD17B6 | HSD17B2 | PPARG | CYP2B6 | UGT1A6 | G6PC | APOB | DGAT1 | DGAT1 | CYP2B6 | PPARG | AGMO |
|  | SRD5A3 | PCSK9 | DGAT1 | DGAT1 | CYP2C9 | APOB | PPARG | CYP2B6 | CYP2B6 | LPL | LPL | PRKG2 |
|  | SRD5A1 | FABP5 | UGT1A6 | UGT1A6 | HPGDS | PPARG | PLA2G1B | HPGDS | HPGDS | EPT1 | EPT1 | APOC1 |
|  | UGT1A10 | CERS6 | UGT1A8 | UGT1A8 | CYP1A2 | SCD5 | DGAT1 | CYP2C9 | APOB | PLA2G1B | PLA2G1B | SOCS3 |
|  | AKR1C1 | SGMS2 | HPGDS | HPGDS | LPL | FA2H | PON1 | APOB | G6PC | HPGDS | HSD17B6 | MMP1 |
|  | HSD17B2 | CYP3A7 | CYP2C9 | CYP2C9 | CYP1A1 | HPGDS | CETP | UGT1A6 | HSD17B6 | APOB | HPGDS | CETP |
