# Supplementary material for: Lipid metabolism gene-wide profile and survival signature of lung adenocarcinoma
Source: Lipids Health Dis. 2020 Oct 13;19:222. doi: 10.1186/s12944-020-01390-9 (PMC7557101; doi:10.1186/s12944-020-01390-9)
Supplement: Supplementary file 4 — Additional file 4: Table S2. Correlation analysis of expression levels of hub genes in LUAD by GEPIA. [file 12944_2020_1390_MOESM4_ESM.docx]

Table S2. Correlation analysis of expression levels of hub genes in LUAD by GEPIA.

|  | R | *P* |
| --- | --- | --- |
| LPL - UGT1A6 | −0.04 | 0.44 |
| LPL - HPGDS | 0.02 | 0.67 |
| LPL - DGAT1 | 0.15 | <0.01* |
| LPL - INS | −0.02 | 0.61 |
| LPL - CYP2C9 | −0.02 | 0.62 |
| UGT1A6 - HPGDS | −0.11 | 0.02* |
| UGT1A6 - DGAT1 | −0.03 | 0.45 |
| UGT1A6 - INS | −0.02 | 0.73 |
| UGT1A6 - CYP2C9 | 0.01 | 0.82 |
| HPGDS - DGAT1 | −0.09 | <0.05* |
| HPGDS - INS | −0.01 | 0.86 |
| HPGDS - CYP2C9 | 0.02 | 0.62 |
| DGAT1 - INS | 0.05 | 0.29 |
| DGAT1 - CYP2C9 | 0.07 | 0.11 |
| INS - CYP2C9 | <0.01 | 0.99 |

* There was a significant correlation between the expression levels of two genes in LUAD.
