# Supplementary figures and images for: Lipid metabolism gene-wide profile and survival signature of lung adenocarcinoma
Source: Lipids Health Dis. 2020 Oct 13;19:222. doi: 10.1186/s12944-020-01390-9 (PMC7557101; doi:10.1186/s12944-020-01390-9)

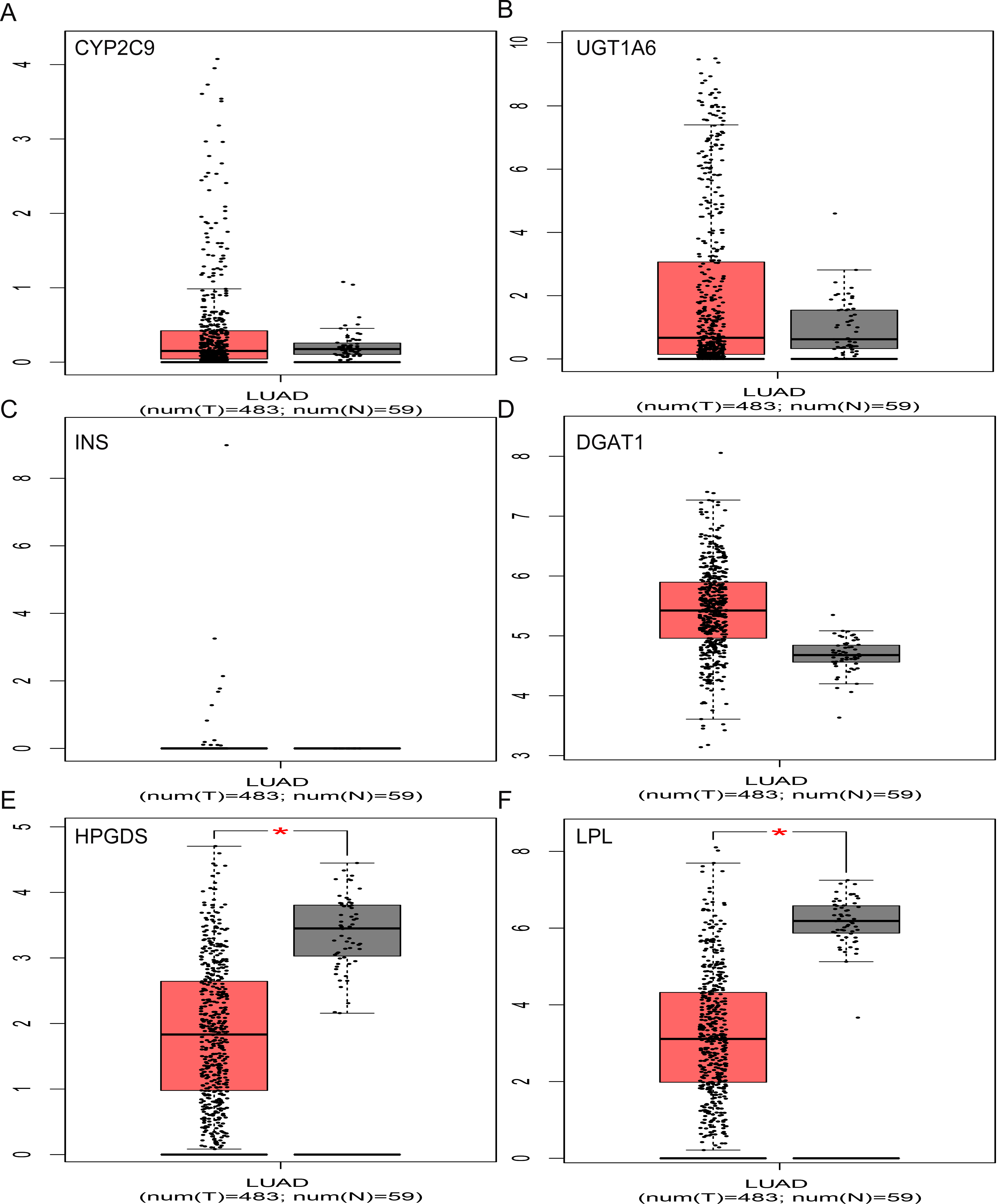

Supplement: Supplementary file 5 — Additional file 5: Figure S1. Expression level analysis of 6 hub genes in GEPIA databases. Red and gray represent cancer and normal, respectively. (A) CYP2C9, (B) UGT1A6, (C) INS, (D) DGAT1, (E) HPGDS, and (F) LPL. * P < 0.05. [file 12944_2020_1390_MOESM5_ESM.tif]

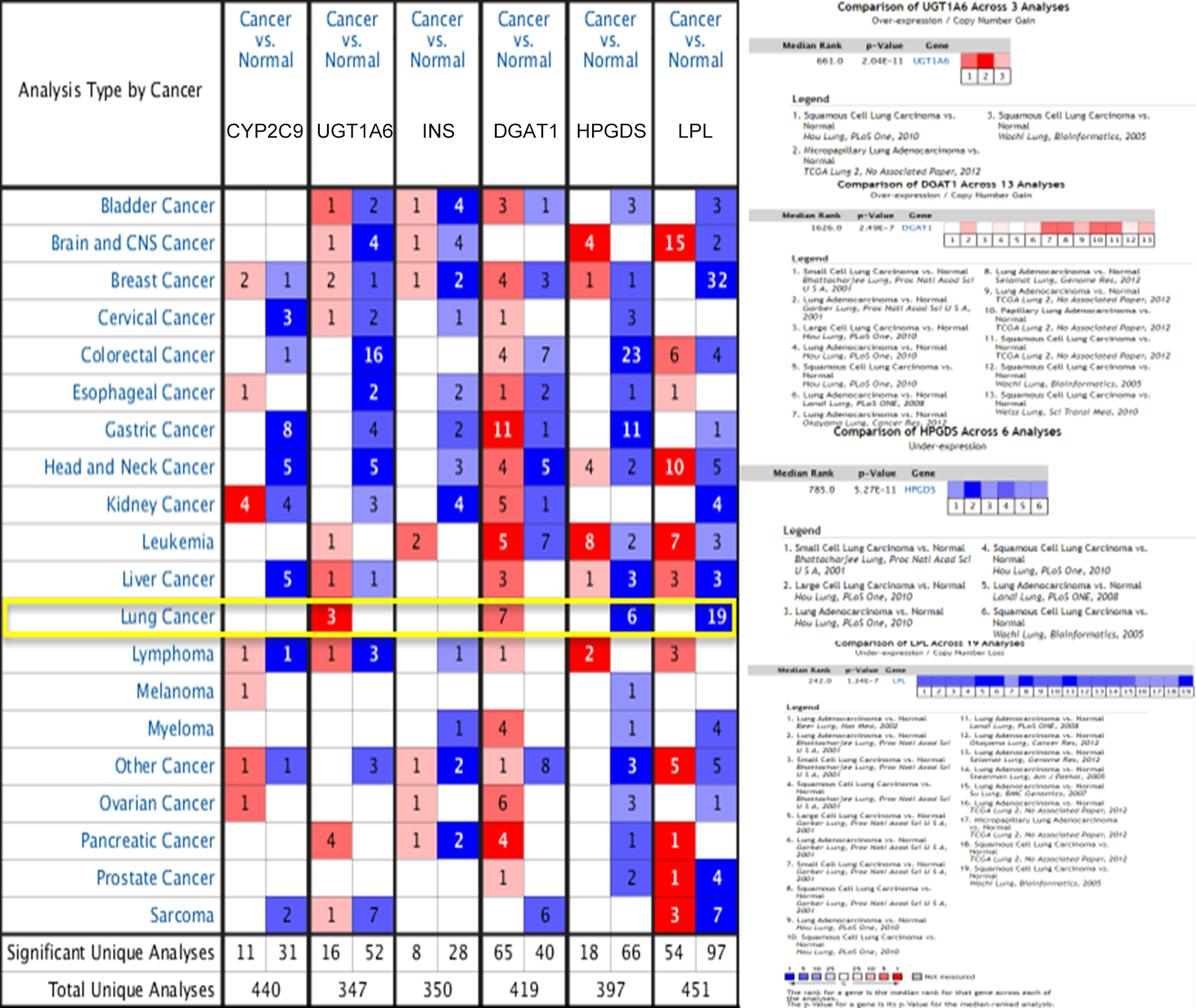

Supplement: Supplementary file 6 — Additional file 6: Figure S2. Meta-analysis of 6 hub genes of lung cancer in ONCOMINE databases. [file 12944_2020_1390_MOESM6_ESM.tif]

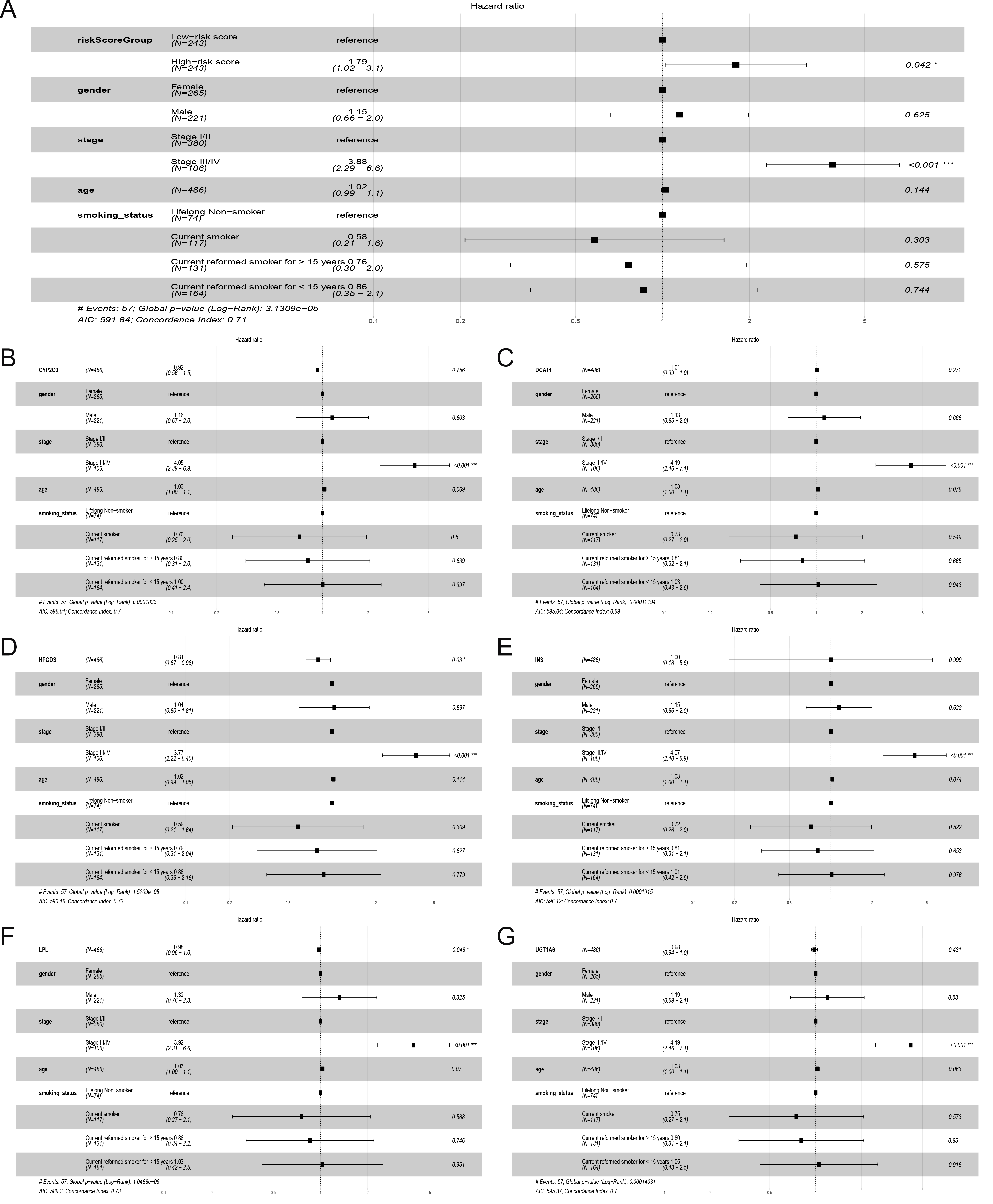

Supplement: Supplementary file 7 — Additional file 7: Figure S3. Forrest plot of the multivariate Cox regression analyzis in TCGA-LUAD. (A) Risk score, (B) CYP2C9, (C) UGT1A6, (D) INS, (E) DGAT1, (F) HPGDS, and (G) LPL. [file 12944_2020_1390_MOESM7_ESM.tif]
